# Genome anatomy of the gastrointestinal pathogen, Vibrio parahaemolyticus of crustacean origin

**DOI:** 10.1186/1757-4749-5-37

**Published:** 2013-12-11

**Authors:** Suma Tiruvayipati, Subha Bhassu, Narender Kumar, Ramani Baddam, Sabiha Shaik, Anil Kumar Gurindapalli, Kwai Lin Thong, Niyaz Ahmed

**Affiliations:** 1 Institute of Biological Sciences, Faculty of Science, University of Malaya, Kuala Lumpur, Malaysia; 2 Pathogen Biology Laboratory, Department of Biotechnology and Bioinformatics, School of Life Sciences, University of Hyderabad, Hyderabad, India; 3 Center of Biotechnology for Agriculture (CEBAR), University of Malaya, Kuala Lumpur, Malaysia; 4 Laboratory of Biomedical Science and Molecular Microbiology, UMBIO Research Cluster, University of Malaya, Kuala Lumpur, Malaysia

**Keywords:** *Vibrio parahaemolyticus*, Genomics, Malaysia, Seafood, Comparative genomics

## Abstract

*Vibrio parahaemolyticus*, an important human pathogen, is associated with gastroenteritis and transmitted through partially cooked seafood. It has become a major concern in the production and trade of marine food products. The prevalence of potentially virulent and pathogenic *V. parahaemolyticus* in raw seafood is of public health significance. Here we describe the genome sequence of a *V. parahaemolyticus* isolate of crustacean origin which was cultured from prawns in 2008 in Selangor, Malaysia (isolate PCV08-7). The next generation sequencing and analysis revealed that the genome of isolate PCV08-7 has closest similarity to that of *V. parahaemolyticus* RIMD2210633. However, there are certain unique features of the PCV08-7 genome such as the absence of TDH-related hemolysin (TRH), and the presence of HU-alpha insertion. The genome of isolate PCV08-7 encodes a thermostable direct hemolysin (TDH), an important virulence factor that classifies PCV08-7 isolate to be a serovariant of O3:K6 strain. Apart from these, we observed that there is certain pattern of genetic rearrangements that makes *V. parahaemolyticus* PCV08-7 a non-pandemic clone. We present detailed genome statistics and important genetic features of this bacterium and discuss how its survival, adaptation and virulence in marine and terrestrial hosts can be understood through the genomic blueprint and that the availability of genome sequence entailing this important Malaysian isolate would likely enhance our understanding of the epidemiology, evolution and transmission of foodborne Vibrios in Malaysia and elsewhere.

## Background

*Vibrio parahaemolyticus* inhabits the estuarine, marine and brackish water ecosystems. It is an important human pathogen associated with gastroenteritis linked to contaminated seafood consumption. Since this species is abundant in marine products, it has become a significant concern in the production and trade of seafood worldwide [1]. In Southeast Asian countries, including Malaysia, virulent *V. parahaemolyticus* in raw seafood have been reported [2,3]. Numerous cases of *V. parahaemolyticus* infection were reported in North America, South East Asia and Japan including some places in East Asia [4-10] giving the illness a pandemic status affecting thousands of people. Thus, the prevalence of pathogenic Vibrios in seafood is of public health concern and is an open ended issue.

The pathogenic *V. parahaemolyticus* strains are differentiated from non-pathogenic ones by their ability to cause beta-haemolysis on Wagatsuma agar, an activity known as ‘Kanagawa phenomenon’. This effect is mediated by the activity of thermostable direct hemolysin (TDH) encoded by the *tdh* genes [8]. A pandemic clone of *V. parahaemolyticus* can broadly be defined as the one that is positive for TDH and exhibits the Kanagawa phenomenon [10].

*V. parahaemolyticus* strains are classified based on the types and variants of their O antigen and flagellar antigen (K). There are 13 O-serogroups and 71 K antigens and various combinations of these give rise to a wide variety of serovars which have been recognized as the causative agents of the disease. A clone of serovar O3:K6 has recently emerged and was associated with outbreaks in India and Japan [7]. Frequent recombination events that promote clonal diversification suggest a scenario whereby a subset of O3:K6 strains might continue to evolve [11]. Consequently, different groups of related O3:K6 clonal strains have now been globally disseminated in Asia, North and South America, Africa and Europe [7].

The genomes of *V. parahaemolyticus* strains are said to have undergone a number of recombination events that could have been the reason for serotype conversion from O3:K6 to O4:K68 [12]. Regions of recombination likely involve a genetic element larger than the gene clusters encoding O and K-antigens. More than 20 serovariants which include O3:K6, O4:K68, O1:K25, O6:K18 and O1:KUT [13,14] emerged from an original pandemic strain, O3:K6. The pandemic group of these bacteria has evolved through a number of deletions, substitutions and acquisitions of regions primarily corresponding to TDH or a TDH-related hemolysin (TRH). It is the presence of either of these two virulence factors that confer potential to cause gastroenteritis in human populations. The pandemic clone is said to have emerged from a pre-pandemic clone which was positive for TRH and negative for TDH genes and harbored a new sequence of toxR (GS-PCR). The intermediate clone is described as being negative for both TRH and TDH, but positive for GS-PCR.

It has been observed that *V. parahaemolyticus* contains two chromosomes; *V. parahaemolyticus* RIMD2210633 has 3.2 Mb and 1.8 Mb of genome sizes for chromosome1 and 2 respectively [15]. There are several *V. parahaemolyticus* genomes which have been sequenced and deposited in Genbank as whole genomes or shotgun submissions (WGS) and sequence read archives (SRA). The only fully annotated submissions entail *V. parahaemolyticus* RIMD2210633 and *V. parahaemolyticus* BB220P*.* The *V. parahaemolyticus* RIMD2210633 genome harbors a Type III secretion system as a central virulence factor which is found in most diarrhea-causing bacteria [15]. As mentioned above, many studies link to the evolutionary aspects of the present pandemic clone formed from a pre-pandemic clone with a drastic change in its gene content i.e., the evolution from a TDH negative/TRH positive to a TDH positive/TRH negative strain and the occurrence of several serovariants in the *V. parahaemolyticus* species. The present isolate (*V. parahaemolyticus* PCV08-7) has been recovered from seafood (prawn) in 2008 which were purchased from a wet market in Selangor, Malaysia.

The main purpose of this study was to analyze the PCV08-7 genome that originates from Malaysia, a large peninsular as well as archipelagic country having a thriving seafood business and that it experiences several food borne outbreaks each season. Unfortunately, there are no markers based on native genome(s) to guide detection of *V. parahaemolyticus* in wet market, in the aquaculture farms and from human excreta and blood. We hope that this genome sequence will be helpful in identifying markers relevant in diagnostic development and molecular epidemiology/transmission dynamics of this significant bacterium in Malaysia and elsewhere.

## Methods

### Source, isolation and culture of V. parahaemolyticus PCV08-7

The *V. parahaemolyticus* PCV08-7 (VPPCV08-7) isolate was identified and characterized by obtaining pure cultures on selective media followed by analysis through biochemical tests, Analytical Profile Index (API) tests and genetic confirmation by PCR. The bacterial culture was maintained by streak plate on a Thiosulfate-Citrate-Bile-Sucrose (Difco, France) agar plates. After incubation at 37°C for 21 – 24 hr, characteristic bacterial colonies appeared with blue-green colored boundaries. An isolated bacterial colony was cultured in Luria-Bertani (LB) broth with 2% Sodium Chloride (NaCl) and incubated overnight at 37°C for 16 – 18 hr. This bacterial culture was further maintained as glycerol stocks at -80°C in 20% glycerol. The genomic DNA was isolated from a pure, single colony. The bacterial identity was confirmed by sequence analysis of the 16S rRNA.

### Genomic DNA isolation and Next-Generation Sequencing

The genomic DNA was isolated using Qiagen DNeasy Blood & Tissue kit (Qiagen, Germany) and the genome sequence was determined by Illumina genome analyzer at the Genotypic Technology Pvt. Ltd. Bengaluru, India (GA2x, pipeline version 1.6). The sequencing data comprised of 100 bp paired-end reads with an insert size corresponding to approximately 240 bp. The genome coverage obtained was approximately about 80X with per base quality of reads in a range of 25 – 40. A total of 3.8 million reads were generated. Bioinformatics analysis was carried out with the help of protocols, algorithms and scripts developed, customized and tested in Ahmed Labs.

### Assembly and alignment

Various strategies were applied to resolve the difficulties in dealing with the two chromosomes to be assembled from the sequence reads. The following main approaches were adopted:

1. *Velvet*[16]: Contigs were generated using the sequence reads which consisted of information from both the chromosomes of the isolate PCV08-7. This was checked by manually comparing contigs against the NCBI database by BLAST to check the highest similarity hit. *V. parahaemolyticus* RIMD2210633 was found to be the closest match in each search. The contigs showed unique hits to chromosome 1 (CHR1) and chromosome 2 (CHR2) as well as few common hits at both the chromosomes. The strategy of using the contigs together representing a whole genome (i.e., CHR1 and CHR2 together) or using the contigs separately as CHR1 and CHR2 was found to be challenging for further analysis to assemble them separately into two chromosomal sequences.

2. *OSLAY*[17]: All the contigs were compared against both the chromosomes of the genome of RIMD2210633 individually and were then used to form supercontigs for both the chromosomes separately. This procedure was found to be problematic as the supercontig files generated from CHR1 and CHR2 (separately) revealed that the preliminary contigs mapped to sequences in both the supercontig files. This was perhaps due to the input file comprising assembled whole genome contigs used against CHR1 and CHR2. The second strategy under OSLAY was to attach CHR1 and CHR2 of the reference genome RIMD2210633 as follows: CHR1 and CHR2 were concatenated (as a ‘whole genome stretch’) and then further used as one full length single sequence. Using this whole genome stretch for BLAST analysis, supercontigs were generated using Velvet contigs and the BLAST results. This also eventually proved inefficient since the supercontigs contained some sequences with several ‘N’ representing a gap in this case and such supercontigs had to be sorted to their own positions on the genome.

3. *SSPACE*[18]: Scaffolding was performed on velvet assembled contigs. As explained above, scaffolds were obtained separately from both CHR1 and CHR2 as well as with the whole genome stretch. All the scaffolds were then BLAST analyzed against both CHR1 and CHR2 of the reference genome individually, as well as at the level of the whole genome stretch. The difficulty faced with scaffolding was similar to that of OSLAY. Hence, the option of separately identifying the scaffolds with respect to CHR1 and CHR2 and dealing with them separately remained a problem.

4. *Mauve*[19]: Velvet assembled contigs were used at this step and exported as sorted contigs by performing an alignment against the whole genome stretch. The results obtained as aligned sorted contigs were taken through a stand-alone BLAST protocol against the whole genome of RIMD2210633. Then the BLAST results were carefully checked for their positions corresponding to both CHR1 and CHR2. The contigs were carefully divided as belonging to CHR1 and CHR2 sequences of PCV08-7 draft genome. The issues faced here were limited to identifying and dealing with the sequences other than those present in the contigs, but which were common to both RIMD2210633 and PCV08-7 genomes. While working on the above strategies, BWA alignment [20] was performed using sequence reads against the whole genome stretch of VPRIMD2210633. Using SAMTOOLS [21] a *.sam* file was generated with which the whole genome of RIMD2210633/FASTA sequence was loaded on Tablet viewer [22] to manually inspect the presence of common genes and to position the draft genome of PCV08-7.

**Figure 1:**
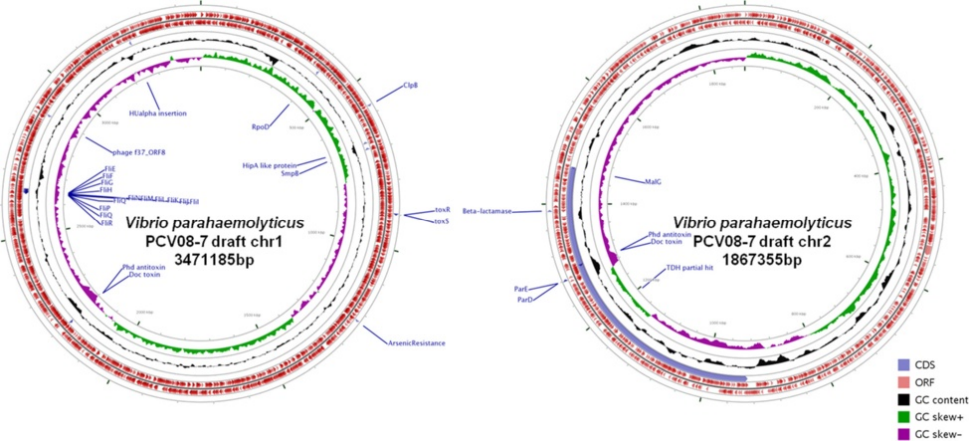
**Circular view of *Vibrio parahaemolyticus *PCV08-7 draft genome.** Diagrammatic representation of major genes carried by the two chromosomes of *Vibrio parahaemolyticus* PCV08-7 genome using CGview [25].

The sequencing reads obtained by us were primarily passed through a quality control step using FASTX toolkit [23] to obtain high quality reads free from adaptor and primer contamination which was further standardized to an optimal parameter p value of 70. High quality reads thus obtained were assembled de-novo [22,23] using the Velvet assembly tool which produced 83 contigs with a hash length optimized to 71. These contigs were used to run OSLAY to form supercontigs with the reference genome RIMD2210633. Alignment of the reads against the reference genome was performed using BWA. The pre-assembled reads were also formed into scaffolds using SSPACE. Perl scripts written in house and modified after Baddam *et al.*[24] were used to re-order the contigs, supercontigs and scaffolds into their individual files. These approaches were put together to finalize the draft genome of *V. parahaemolyticus* PCV08-7 (Figure 1).

## Results and discussion

### Genome assembly

The 100 bp paired end reads were assembled using Velvet assembly tool that effectively utilized approximately 3.7 million reads. The N50 value observed was 261989 bp. The contig with the maximum length was 704232 bp and the total number of bases in the genome were 5184164 bp. The genome was artificially closed.

The genomes with multiple chromosomes pose technical difficulties during assembly. It is a known fact that Vibrios – *V. cholerae, V. parahaemolyticus* and *V. vulnificus* contain two circular chromosomes [26]. The reference genome used in this study, *V. parahaemolyticus* RIMD2210633 also consists of two chromosomes [13]. As studied previously [13], the origin of replication in chromosome 1 with the presence of *dnaA* gene shows its similarity to many genomes of prokaryotic origin and the origin of replication of chromosome 2 shows homology with that present on *V. cholerae* chromosome 2. The identification of distinct replication sites is of utmost importance for assembling bacterial genomes with two chromosomes which in the case of *V. cholerae* have been studied earlier [27]. Previous studies explain need for a more accurate procedure to handle data to correctly assemble two chromosomes and assign gene locations. The reads were assembled into a total of 83 contigs which were separated based on the assemble strategy as explained in the materials and methods section. Dealing with the present data, we observed that many of the genes of significant virulence or fitness importance were located on the chromosomes rather than showing any significant homology to the *Vibrio* plasmids. The presence of the Phd-Doc toxin antitoxin gene in our genome makes it interesting as the antitoxin gene has been previously reported related to plasmids [28] while a recent study [29] described its occurrence on the chromosome of *Vibrio* species. However, we agree that the exact source of these genes can be mapped only when the plasmids will be sequenced and or analyzed separately.

### Genome statistics and annotation

The draft assembled genome was annotated using the RAST server [30]. Statistics of the *V. parahaemolyticus* PCV08-7 draft genome were derived using Artemis [31], RNAmmer [32] and tRNAscanSE [33]: the sizes of chromosome 1 and chromosome 2 of the isolate were 3471185 bp and 1867355 bp respectively with G + C content of 45.35%. The tRNA and rRNA genes were 102 and 31 for chromosome 1, and 13 and 3 for chromosome 2, respectively. The chromosome 1 revealed a coding percentage of 85 with an average gene length of 943 bp while the chromosome 2 had a coding percentage of 86.2 with an average gene length of 950 bp.

**Figure 2:**
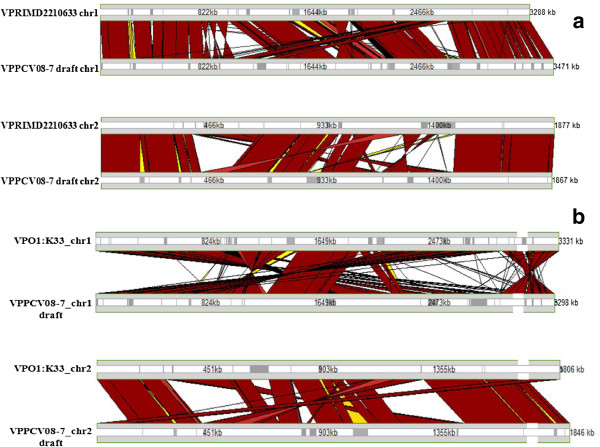
**Alignment of the genome of strain RIMD2210633 against that of isolate PCV08-7 and strain O1:K33. (a)** Comparison of chromosomes of strain RIMD2210633 (VPRIMD2210633 chr1, VPRIMD2210633 chr2) with the draft chromosomes of PCV08-7 (VPPCV08-7 draft chr1, VPPCV08-7 draft chr2) using M-GCAT. **(b)** Comparison of chromosomes of strain O1:K33 (VPO1:K33 chr1, VPO1:K33 chr2) with the draft chromosomes of PCV08-7 (VPPCV08-7 draft chr1, VPPCV08-7 draft chr2) using M-GCAT.

The alignment of *V. parahaemolyticus* PCV08-7 genome with that of the *V. parahaemolyticus* RIMD2210633 genome using M-GCAT [34] showed visible rearrangements in the sequences of the two chromosomes of PCV08-7 isolate (Figure 2). The chromosome 1 of the draft genome carried phage shock proteins A, B and C, and bacteriophage f237 ORF8. It contained an integrated *tmRNA* gene with the closest element encoding the ribonuclease H. A site-specific recombinase *IntI4* and a gene encoding beta-lactamase were present. The draft genome also revealed genes responsible for fatty acid and amino acid metabolism. An important outer membrane protein *OmpU* was also identified. Genes coding for gyrase B (*gyrB*), HU-alpha insertion and putative sigma factors such as *rpoD, rpoE, rpoS, rpoN and rpoH* were also found in our analysis. The chromosome 2 carried a TDH pathogenicity island with many deletions and substitutions and displayed a *malG* gene on one of the flanking regions of the pathogenicity island. This region also contained genes coding for nutrient uptake and metabolism. We documented the presence of vibrio ferrin receptor *pvuA* and ferrichrome ABC transport *pvuB*, *pvuC*, *pvuD* and *pvuE* encoding genes, and the related *pvsA*, *pvsB*, *pvsC*, *pvsD* and *pvsE* genes. The analysis of the genome further revealed presence of a cobalt-zinc-cadmium resistance protein and a Rhodanese related sulfur transferase (as also present in RIMD2210633 genome) and a lead-cadmium-zinc-mercury transporting ATPase enzyme (as seen in the *V. parahaemolyticus* BB220P genome). Phd antitoxin and Doc toxin [28] which fall under the programmed cell death systems were also uniquely identified. Studies in *E. coli* have shown the presence of a stress related protein *clpB* along with *rpoS* and a few other genes [35] which help cope with stress conditions and help in survival. Our analysis detected the presence of *clpB, rpoS* and *hipA* genes in the present genome as was also seen in the reference genome of RIMD2210633. There were two types of Type III secretion systems observed in *V. parahaemolyticus* RIMD2210633 [36]; T3SS1 and T3SS2. Our genome analysis remains open ended with respect to the presence of such type III secretion systems.

### Identification of novel gene content and comparative analysis

**Figure 3:**
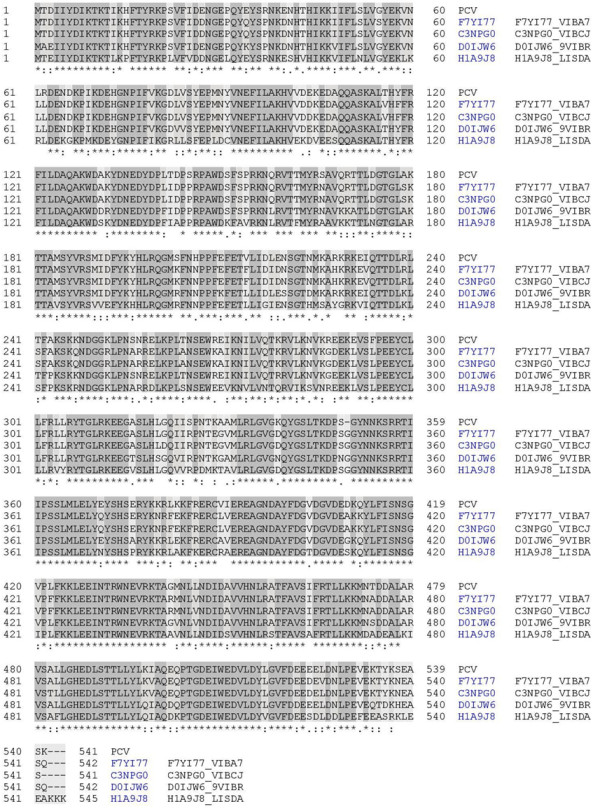
**Alignment of a unique PCV08-7 protein sequence similar to *Photobacterium damselae subsps. damselae*.** A unique sequence from PCV08-7 genome showed similarity with putative uncharacterized proteins of *V. anguillarum* 775 (F7YI77), *V. cholera* MJ-1236 (C3NPG0) and *Vibrio sp.* RC586 (D0IJW6) and similarity to a phage integrase of *Photobacterium damselae subsps. damselae* (H1A9J8).

Our genome analysis revealed some unique sequences which have good similarity to hypothetical proteins of other Vibrio species such as *Vibrio anguillarum* and *Vibrio cholerae*. A 6315 bp nucleotide sequence showed identity to a *V. anguillarum* hypothetical protein and a *V. cholera* hypothetical protein on NCBI-BLASTN. One of the coding proteins in this stretch revealed similarity to the annotated phage integrase encoding gene of *Photobacterium damselae subsp. damselae* plasmid pAQU1 DNA (Figure 3). A parD gene (antitoxin to parE) was also found which showed closer identity to other *Vibrio* species such as *Vibrio vulnificus*, *Vibrio mimicus* and *Vibrio orientalis*. parD when aligned against *V. vulnificus* and *V. mimicus* revealed an identity of 76 bp out of 80 bp (95%) (e-value 2e-48) and with *V. orientalis* an identity of 72 bp out of 80 bp (90%) (e-value 2e-45) on NCBI-BLASTN. A few newer hypothetical proteins with no reported annotation were identified. The genome also contained a gene relevant to arsenic resistance, possibly important in the adaptation of the bacterium to a high arsenic environment. Our analysis of the genome revealed presence of a partially similar sequence of TDH Pathogenicity Island, as compared to *V. parahaemolyticus* RIMD2210633. This island revealed genetic instability due to various insertion/deletion and substitution events we documented. The presence of toxS and toxR genes was also observed.

**Figure 4:**
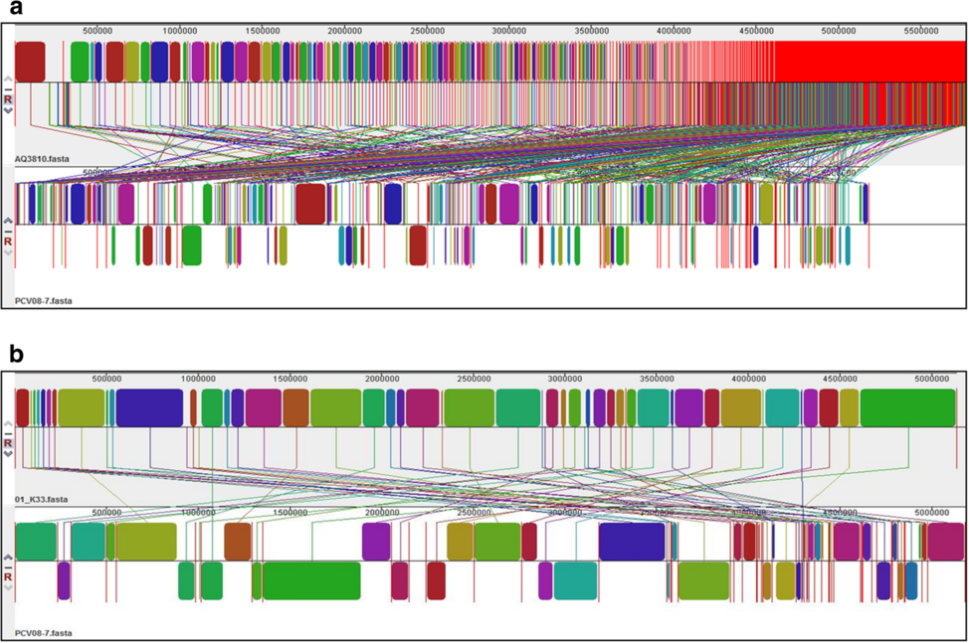
**(a) *V. parahaemolyticus *AQ3810 alignment against PCV08-7 genome: Concatenated chromosome 1 and chromosome 2 of *V. parahaemolyticus *AQ3810 (AQ3810.fasta) against *V. parahaemolyticus *PCV08-7 (PCV08-7.fasta) (b) *V. parahaemolyticus *O1:K33 alignment against PCV08-7.** Concatenated chromosome 1 and chromosome 2 of *V. parahaemolyticus* O1:K33 (O1_K33.fasta) against *V. parahaemolyticus* PCV08-7 (PCV08-7.fasta).

**Figure 5:**
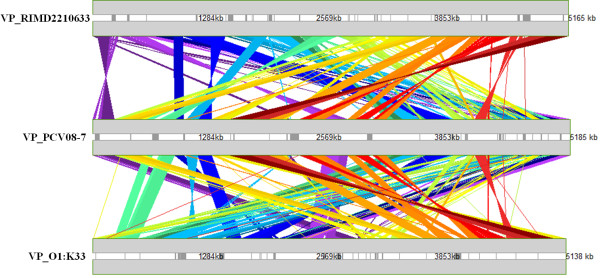
**Comparison of whole genome sequences of strains RIMD2210633, PCV08-7 and O1:K33.** Alignment of complete genomes of *V. parahaemolyticus* RIMD2210633, *V. parahaemolyticus* PCV08-7 and *V. parahaemolyticus* O1:K33, showing PCV08-7 being more similar to RIMD2210633.

The old pandemic O3:K6 strain of *V. parahaemolyticus* is said to have gained gene clusters VPaI1-VPaI7 [37] to develop into a new pandemic clone of which VPaI4-VPaI6 are said to be putative virulence factors and may be potential pathogenicity islands. These regions are said to carry along with them a type VI secretion system (VP1386-1420). Our PCV08-7 genome analysis revealed that only one cluster, VPaI2, was detected completely, whereas VPaI3 and VPaI7 were partially present (Table 1). This perhaps shows that our strain could be possibly a new serovariant of a non-pandemic O3:K6 strain like the *V. parahaemolyticus* AQ3810 [8]. While variability of different gene clusters (Table 1) portrays a probably novel serovariant of *V. parahaemolyticus* with the presence of ribonuclease H encoding element (previously thought to be present only in *V. parahaemolyticus* RIMD2210633 and absent in *V. parahaemolyticus* AQ3810 [12]). A further comparative study between the *V. parahaemolyticus* PCV08-7 and the non-pandemic *V. parahaemolyticus* AQ3810 (O3:K6 strain) and the newest *V. parahaemolyticus* O1:K33 (trh+/ tdh + genotype) strain showed that *V. parahaemolyticus* PCV08-7 has more genetic relatedness towards a trh+/ tdh + strain (Figure 4). But, alignments of the *V. parahaemolyticus* PCV08-7 contig data against the *V. parahaemolyticus* O1:K33 and *V. parahaemolyticus* RIMD2210633 (Figure 2) strains show that it is closer to O3:K6 serotype (Figure 5).

**Table 1:** **Table representing pathogenicity related clusters and other VP clusters in** ***V. parahaemolyticus*** **PCV08-7: (1) pathogenicity related clusters (VPaI1-VPaI7) in the genome of strain RIMD2210633 that signify it to be a pandemic O3:K6 strain and their presence or absence in the genome of PCV08-7 isolate, (2) various other VP clusters and their occurrence in the genome of PCV08-7**

**(1) *Vibrio parahaemolyticus *** **RIMD2210633**	***V. parahaemolyticus*** **PCV08-7**
VPaI1 (VP0380-0403)	Absent
VpaI2 (VP0635-0643)	Present
VpaI3 (VP1071-1094)	Partially present
VpaI4 (VP2131-2144)	Absent
VpaI5 (VP2900-2910)	Absent
VpaI6 (VPA1254-1270)	Absent
VpaI7 (VP1312-1398)	Present
Type VI secretion system (VP1386-1420)	Absent
**(2) Other VP clusters**	***V*. *parahaemolyticus*** **PCV08-7**
VP1355-1368	Partially present
VPA0074-0089	Present
VPA0713-0732	Present
VPA1194-1210	Present

From the above thesis, it becomes probably apparent that the genome of *V. parahaemolyticus* PCV08-7 meaningfully adds to the battery of important genomic sequences representing enteropathogenic bacteria. The genome of an arthropod derived, foodborne *Vibrio* should be important to understand adaptation to a crustacean host and a human host.

### Epilogue and future directions

A first account of the genome of *V. parahaemolyticus* PCV08-7 has been presented. The draft genome and its annotation as described would be able to explain the lifestyle of pathogenic *Vibrio* species. The experience of assembling this genome and the difficulties associated with separating the data with respect to two chromosomes would certainly be helpful to the community in the follow-up studies. Further, a host of new molecular markers as gleaned by our analysis would be relevant in the diagnostic development and molecular epidemiology. The present genome and the ensuing comparative genomics would be able to rekindle our thoughts on the survival and virulence as well as transmission potentials of *V. parahaemolyticus* and also on their adaptation to different hosts and the niches thereof. Our results clearly reveal a significantly novel gene content which could presumably have been acquired through a horizontal gene transfer mechanism. Our analysis revealed the presence of not only the conserved genomic regions among different *V. parahaemolyticus* bacteria, but also dissects some of the unique sets of genes that hold relevance to virulence. We propose to finish and polish the genome in the near future also with the help of further coverage using alternative sequencing platforms and by employing a hybrid assembly approach. Also, it will be possible to determine the true extent of the diversity of *V. parahaemolyticus* strains obtained from seafood as compared to those isolated from human cases. Such a diversity analysis would focus on 1) genomic coordinates relevant to colonization of and adaptation to different hosts in different ecosystems; 2) genome dynamics relative to bacterial fitness shaping over time and with transmission across different hosts; and 3) profile of genomic rearrangements including additive and reductive genome evolution and their significance in the evolution of pathogenic *Vibrio* species. Presently, the epidemiology of *V. parahaemolyticus* infection in resource-poor countries largely entails a classical serology concocted with guess work as to the type of strain involved and its source. Our genomic data would hopefully contribute to this situation also.

## Availability of supporting data

The *Vibrio parahaemolyticus* PCV08-7 whole genome shotgun project was deposited in Genbank under the accession AOCL00000000. The version described in this paper is the first version, AOCL01000000. This consists of sequences from AOCL01000000 – AOCL01000083 (http://www.ncbi.nlm.nih.gov/nuccore/AOCL00000000).

## Competing interests

NA and TKL are the editors of Gut Pathogens.

## Authors’ contributions

NA and SB: Designed and supervised the study and written and edited the manuscript, TS: performed genomic DNA preparation, sequencing analysis, annotation and comparative genomics, AKG: performed initial bioinformatics analysis, RB and SS: provided tools and IT support for the study, NK: contributed to quality control of the NGS data and assembly. TKL: isolated and maintained the strain and provided inputs on lifestyle and evolution of the organism. All authors read and approved the final manuscript.

## References

[B1] DePaola A Nordstrom JL Dalsgaard A Forslund A Oliver J Bates T Bourdage KL Gulig PA Analysis of Vibrio vulnificus from market oysters and septicemia cases for virulence markers Appl Environ Microbiol 2003 5 7 4006 4011 10.1128/AEM.69.7.4006-4011.2003 12839775 PMC165197

[B2] Sujeewa AKW Norrakiah AS Laina M Prevalence of toxic genes of Vibrio parahaemolyticus in shrimps (Penaeus monodon) and culture environment International Food Research Journal 2009 5 89 95

[B3] Paydar M Teh CS Thong KL Prevalence and characterisation of potentially virulent Vibrio parahaemolyticus in seafood in Malaysia using conventional methods, PCR and REP-PCR Food Control 2013 5 13 18 10.1016/j.foodcont.2012.11.034

[B4] Guidelines for national human immunodeficiency virus case surveillance, including monitoring for human immunodeficiency virus infection and acquired immunodeficiency syndrome. Centers for Disease Control and Prevention MMWR Recomm Rep: Morbidity and mortality weekly report Recommendations and reports/Centers for Disease Control 1999 5 RR-13 1 27 29–31 10632297

[B5] Bag PK Nandi S Bhadra RK Ramamurthy T Bhattacharya SK Nishibuchi M Hamabata T Yamasaki S Takeda Y Nair GB Clonal diversity among recently emerged strains of Vibrio parahaemolyticus O3:K6 associated with pandemic spread J Clin Microbiol 1999 5 7 2354 2357 10364615 10.1128/jcm.37.7.2354-2357.1999 PMC85163

[B6] Nair GB Hormazabal JC The Vibrio parahaemolyticus pandemic Revista chilena de infectologia: organo oficial de la Sociedad Chilena de Infectologia 2005 5 2 125 130 15891792 10.4067/s0716-10182005000200002

[B7] Nair GB Ramamurthy T Bhattacharya SK Dutta B Takeda Y Sack DA Global dissemination of Vibrio parahaemolyticus serotype O3:K6 and its serovariants Clin Microbiol Rev 2007 5 1 39 48 17223622 10.1128/CMR.00025-06 PMC1797631

[B8] Nishibuchi M Kaper JB Thermostable direct hemolysin gene of Vibrio parahaemolyticus: a virulence gene acquired by a marine bacterium Infect Immun 1995 5 6 2093 2099 7768586 10.1128/iai.63.6.2093-2099.1995 PMC173271

[B9] Okuda J Ishibashi M Hayakawa E Nishino T Takeda Y Mukhopadhyay AK Garg S Bhattacharya SK Nair GB Nishibuchi M Emergence of a unique O3:K6 clone of Vibrio parahaemolyticus in Calcutta, India, and isolation of strains from the same clonal group from Southeast Asian travelers arriving in Japan J Clin Microbiol 1997 5 12 3150 3155 9399511 10.1128/jcm.35.12.3150-3155.1997 PMC230139

[B10] Han H Wong HC Kan B Guo Z Zeng X Yin S Liu X Yang R Zhou D Genome plasticity of Vibrio parahaemolyticus: microevolution of the ‘pandemic group’ BMC genomics 2008 5 570 10.1186/1471-2164-9-570 19038058 PMC2612023

[B11] Gonzalez-Escalona N Martinez-Urtaza J Romero J Espejo RT Jaykus LA DePaola A Determination of molecular phylogenetics of Vibrio parahaemolyticus strains by multilocus sequence typing J Bacteriol 2008 5 8 2831 2840 10.1128/JB.01808-07 18281404 PMC2293261

[B12] Chen Y Stine OC Badger JH Gil AI Nair GB Nishibuchi M Fouts DE Comparative genomic analysis of Vibrio parahaemolyticus: serotype conversion and virulence BMC Genomics 2011 5 294 10.1186/1471-2164-12-294 21645368 PMC3130711

[B13] Chowdhury NR Chakraborty S Ramamurthy T Nishibuchi M Yamasaki S Takeda Y Nair GB Molecular evidence of clonal Vibrio parahaemolyticus pandemic strains Emerg Infect Dis 2000 5 6 631 636 10.3201/eid0606.000612 11076722 PMC2640929

[B14] Chowdhury NR Stine OC Morris JG Nair GB Assessment of evolution of pandemic Vibrio parahaemolyticus by multilocus sequence typing J Clin Microbiol 2004 5 3 1280 1282 10.1128/JCM.42.3.1280-1282.2004 15004094 PMC356825

[B15] Makino K Oshima K Kurokawa K Yokoyama K Uda T Tagomori K Iijima Y Najima M Nakano M Yamashita A Genome sequence of Vibrio parahaemolyticus: a pathogenic mechanism distinct from that of V cholerae Lancet 2003 5 9359 743 749 10.1016/S0140-6736(03)12659-1 12620739

[B16] Zerbino DR Birney E Velvet: algorithms for de novo short read assembly using de Bruijn graphs Genome Res 2008 5 5 821 829 10.1101/gr.074492.107 18349386 PMC2336801

[B17] Richter DC Schuster SC Huson DH OSLay: optimal syntenic layout of unfinished assemblies Bioinformatics 2007 5 13 1573 1579 10.1093/bioinformatics/btm153 17463020

[B18] Boetzer M Henkel CV Jansen HJ Butler D Pirovano W Scaffolding pre-assembled contigs using SSPACE Bioinformatics 2011 5 4 578 579 10.1093/bioinformatics/btq683 21149342

[B19] Darling AE Mau B Perna NT ProgressiveMauve: multiple genome alignment with gene gain, loss and rearrangement PLoS One 2010 5 6 e11147 10.1371/journal.pone.0011147 20593022 PMC2892488

[B20] Li H Durbin R Fast and accurate long-read alignment with Burrows-Wheeler transform Bioinformatics 2010 5 5 589 595 10.1093/bioinformatics/btp698 20080505 PMC2828108

[B21] Li H Handsaker B Wysoker A Fennell T Ruan J Homer N Marth G Abecasis G Durbin R Genome project data processing S: the sequence alignment/Map format and SAMtools Bioinformatics 2009 5 16 2078 2079 10.1093/bioinformatics/btp352 19505943 PMC2723002

[B22] Milne I Bayer M Cardle L Shaw P Stephen G Wright F Marshall D Tablet–next generation sequence assembly visualization Bioinformatics 2010 5 3 401 402 10.1093/bioinformatics/btp666 19965881 PMC2815658

[B23] Taylor J Schenck I Blankenberg D Nekrutenko A Using galaxy to perform large-scale interactive data analyses Curr Protoc Bioinformatics 2007 5 10.5

[B24] Baddam R Kumar N Shaik S Suma T Ngoi ST Thong KL Ahmed N Genome sequencing and analysis of Salmonella enterica serovar Typhi strain CR0063 representing a carrier individual during an outbreak of typhoid fever in Kelantan, Malaysia Gut Pathogens 2012 5 1 20 10.1186/1757-4749-4-20 23234298 PMC3528463

[B25] Stothard P Wishart DS Circular genome visualization and exploration using CGView Bioinformatics 2005 5 537 539 10.1093/bioinformatics/bti054 15479716

[B26] Yamaichi Y Iida T Park KS Yamamoto K Honda T Physical and genetic map of the genome of Vibrio parahaemolyticus: presence of two chromosomes in Vibrio species Mol Microbiol 1999 5 5 1513 1521 10.1046/j.1365-2958.1999.01296.x 10200969

[B27] Egan ES Waldor MK Distinct replication requirements for the two Vibrio cholerae chromosomes Cell 2003 5 4 521 530 10.1016/S0092-8674(03)00611-1 12941279

[B28] McKinley JE Magnuson RD Characterization of the Phd repressor-antitoxin boundary J Bacteriol 2005 5 2 765 770 10.1128/JB.187.2.765-770.2005 15629948 PMC543551

[B29] Guerout AM Iqbal N Mine N Ducos-Galand M Van Melderen L Mazel D Characterization of the phd-doc and ccd Toxin-Antitoxin Cassettes from Vibrio Superintegrons J Bacteriol 2013 5 10 2270 2283 10.1128/JB.01389-12 23475970 PMC3650543

[B30] Aziz RK Bartels D Best AA DeJongh M Disz T Edwards RA Formsma K Gerdes S Glass EM Kubal M The RAST Server: rapid annotations using subsystems technology BMC Genomics 2008 5 75 10.1186/1471-2164-9-75 18261238 PMC2265698

[B31] Rutherford K Parkhill J Crook J Horsnell T Rice P Rajandream MA Barrell B Artemis: sequence visualization and annotation Bioinformatics 2000 5 10 944 945 10.1093/bioinformatics/16.10.944 11120685

[B32] Lagesen K Hallin P Rodland EA Staerfeldt HH Rognes T Ussery DW RNAmmer: consistent and rapid annotation of ribosomal RNA genes Nucleic Acids Res 2007 5 9 3100 3108 10.1093/nar/gkm160 17452365 PMC1888812

[B33] Schattner P Brooks AN Lowe TM The tRNAscan-SE, snoscan and snoGPS web servers for the detection of tRNAs and snoRNAs Nucleic Acids Res 2005 5 Web Server issue W686 W689 15980563 10.1093/nar/gki366 PMC1160127

[B34] Treangen TJ Messeguer X M-GCAT: interactively and efficiently constructing large-scale multiple genome comparison frameworks in closely related species BMC Bioinformatics 2006 5 433 10.1186/1471-2105-7-433 17022809 PMC1629028

[B35] Wang X Wood TK Toxin-antitoxin systems influence biofilm and persister cell formation and the general stress response Appl Environ Microbiol 2011 5 16 5577 5583 10.1128/AEM.05068-11 21685157 PMC3165247

[B36] Park KS Ono T Rokuda M Jang MH Okada K Iida T Honda T Functional characterization of two type III secretion systems of Vibrio parahaemolyticus Infect Immun 2004 5 11 6659 6665 10.1128/IAI.72.11.6659-6665.2004 15501799 PMC523034

[B37] Hurley CC Quirke A Reen FJ Boyd EF Four genomic islands that mark post-1995 pandemic Vibrio parahaemolyticus isolates BMC Genomics 2006 5 104 10.1186/1471-2164-7-104 16672049 PMC1464126

